# Analysis of serum exosomal microRNAs and clinicopathologic features of patients with pancreatic adenocarcinoma

**DOI:** 10.1186/1477-7819-11-219

**Published:** 2013-09-04

**Authors:** Risheng Que, Guoping Ding, Jionghuang Chen, Liping Cao

**Affiliations:** 1 Department of General Surgery, Second Affiliated Hospital, School of Medicine, Zhejiang University, Hangzhou 310009, China

**Keywords:** Blood, microRNA, miR-17-5p, miR-21, Pancreatic adenocarcinoma

## Abstract

**Background:**

Altered expression of serum microRNAs (miRNAs) have been reported to correlate with carcinogenesis and progression of pancreatic adenocarcinoma (PC), but descriptions of serum exosomal miRNAs in PC are still lacking. This study was designed to evaluate serum exosomal miRNA levels in PC patients and to investigate their relationships with clinicopathologic features and prognosis.

**Methods:**

Four miRNAs (miR-17-5p, miR-21, miR-155 and miR-196a) related to PC were selected for examination in our research. Serum miRNA was examined by RT-PCR in a group of 49 patients, including 22 with PCs, 6 with benign pancreatic tumors, 7 with ampullary carcinomas, 6 with chronic pancreatitis and 8 healthy participants. The clinicopathologic data were also collected, and PC patients were classified according to the presence of metastasis, tumor differentiation and advanced stage.

**Results:**

There were low expressions of exosomal miR-155 and miR-196a in serum samples of PC patients when U-6 was used as a control. Serum exosomal miR-17-5p was higher in PC patients than in non–PC patients and healthy participants. High levels of miR-17-5p were significantly correlated with metastasis and advanced stage of PC. The serum exosomal miR-21 level in PC was higher than that in the normal and chronic pancreatitis groups, but was not significantly correlated with PC differentiation and tumor stage.

**Conclusions:**

There were high expressions of serum exosomal miR-17-5p and miR-21 in PC patients. Examination of serum exosomal microRNA is a useful serum biomarker for PC diagnosis other than serum-free microRNA. It is postulated that exosomal miR-17-5p participates in the progression of PC.

## Background

Pancreatic adenocarcinoma (PC) is one of the most malignant cancers, with an overall 5-year survival rate of 3% to 6% [1]. In 2010, an estimated 43,140 people were diagnosed with PC and approximately 36,800 people died as a result of this disease in the United States, making PC the fourth-leading cause of cancer-related deaths in men (after lung, prostate and colorectal cancer) and in women (after lung, breast and colorectal cancer) [2]. Because of a lack of improvement in early detection and desirable treatment strategies, the prognosis of PC patients is extremely poor. Few patients can be cured by surgery [3]. Thus, it is still necessary to search for better diagnostic biomarkers that accurately represent biological characteristics of PC, can be used to screen for early-stage PC and can predict clinical outcomes, which would allow a greater percentage of patients to undergo curative surgery.

MicroRNAs (miRNAs) are small, noncoding RNAs about 22 nt in length that regulate gene expression at the posttranscriptional level and play an important role in cell proliferation, apoptosis and differentiation [4]. The microRNA database (miRBase release 17.0: http://www.mirbase.org/) comprises 1,424 human miRNAs to date [5], many of which were found to correlate with cancer. Among the 1,424 miRNAs, about 100 associated with dysregulated expression in PC have been reported [6]. The expression pattern of miRNAs seems to be a better way than that of mRNA to identify cancer type, because these are more informative during tumor carcinogenesis, differentiation and progression. Several studies have recently shown the aberrant expression of serum miRNAs in PC, which might be useful in early diagnosis of PC. For instance, Bloomston *et al*. [7] identified 21 miRNAs with increased expression and 4 with decreased expression that correctly differentiated PC from benign pancreatic tissue. Fifteen overexpressed and eight underexpressed miRNAs differentiated pancreatic cancer from chronic pancreatitis (CP). *miR-21*, which has been well-described in the literature, has been reported to be strongly overexpressed in PC and to significantly upregulate PDCD4 expression, thus contributing to cell proliferation and invasion, as well as chemoresistance, in patients with PC [8].

Over the past few years, circulating miRNAs have emerged as novel biomarkers for detecting cancer and predicting patients’ prognosis. Despite the findings of previous researchers that more than 100 miRNAs are aberrantly expressed in PC [6,7], descriptions of serum exosomal miRNAs in PC are still lacking, especially regarding the correlation between serum exosomal miRNAs and clinicopathologic features of PC, which is still unknown. In the present study, we examined four miRNAs in the serum exosome sample. The four miRNAs have been well-documented to have high expression levels in the blood of PC patients. We have identified new potential biomarkers for early diagnosis of PC, as well as metastasis and stage prediction. Our results lay the foundation for the development of serum exosomal miRNAs as novel biomarkers for PC detection and also raise provocative questions regarding the potential biological function of exosomal miRNAs in PC progression.

## Methods

### Ethics statement

All participants or their guardians gave their written consent for their serum samples and medical information to be used for scientific research. The research protocol was reviewed and approved by the Research Ethics Committee of Zhejiang University.

### Patients and serum samples

Serum samples were collected from the peripheral veins of 49 patients at the Department of General Surgery, Second Affiliated Hospital of Zhejiang University School of Medicine, including 22 patients with PC, 6 with ampullary carcinoma (AC), 7 with benign pancreatic tumors (BPTs), 6 with CP and 8 healthy participants (HP). All the patients’ serum samples were histologically confirmed, and the HPs were age- and gender-matched with the PC group (Table 1). The serum samples were centrifuged at 3,000 rpm for 10 min and then stored at −80°C.

**Table 1:** Details of patients’ clinical information

**Category**	**Male**	**Female**	**Total**	**Mean age (yr)**
Pancreatic carcinoma	16	6	22	65.3 ± 9.99
Non–pancreatic carcinoma	20	7	27	58.4 ± 13.16
Ampullary carcinoma	5	1	6	40.7 ± 8.99
Benign pancreatic tumor	5	2	7	67 ± 6.19
Chronic pancreatitis	4	2	6	63.8 ± 12.67
Healthy participants	6	2	8	60.3 ± 8.08
Total	31	18	49	61.5 ± 12.22

Patients were divided into a PC group and a non-PC group (HPs and patients with AC, BPT or CP). Pathologic classification was performed according to the International Union Against Cancer (UICC) and American Joint Committee on Cancer (AJCC) TNM staging system for PC established in 2010 [9]. Stages I and II were considered as early stage and stages III and IV as advanced stage. Patients were also classified by tumor differentiation and the presence of metastasis (Table 2). Poorly and poorly to moderately differentiated pancreatic ductal adenocarcinomas (PDACs) were considered as poor differentiation. Well- and well- to moderately differentiated PDACs were assigned to the well–differentiated group. The metastatic conditions of PC were determined on the basis of both imaging features and intraoperative pathological examination. We used an observation period of 3 months in the nonmetastatic patients to exclude potential metastatic cases.

**Table 2:** **Analysis of clinicopathologic features of the primary adenocarcinoma group**^**a**^

**Category**	**Subcategory**	**Male ( *n *)**	**Female ( *n *)**	**Total ( *N *)**	**Mean age (yr)**
TNM stage	Early (I and II)	7	2	9	61.3 ± 9.47
	Advanced (III and IV)	9	4	13	68.1 ± 9.73
PDAC	Poorly differentiated	4	2	6	59.7 ± 11.06
	Well-differentiated	12	4	16	67.9 ± 10.80
Metastasis	Yes	12	6	18	65.4 ± 10.79
	No	4	0	4	64.8 ± 6.24
Total		16	6	22	65.3 ± 9.99

^a^PDAC = pancreatic ductal adenocarcinoma; TNM = tumor, node, metastasis.

### Isolation of serum exosomal miRNAs

First, 1 ml of serum was filtered through a 0.22-μm filter (EMD Millipore, Billerica, MA, USA) to remove cell debris and other cellular organelles, then the filtrate was ultracentrifugated at 10^5^ *g* for 1 h twice. Exosomes were collected from the precipitates, and small RNA was enriched using the *mir*Vana PARIS RNA isolation kit (Ambion, Austin, TX, USA) according to the manufacturer’s protocol. Briefly, 625 μl of cell disruption buffer was added to the precipitate. Next, an equal volume of 2× denaturing solution was added and mixed sufficiently before being mixed with an equal total volume of acid-phenol:chloroform. A *mir*Vana miRNA column was used to collect total RNA. The bound RNA was cleaned with the buffers provided by the manufacturer to remove impurities and eluted in a final volume of 100 μl.

### Screening and verification of circulating miRNAs by RT-PCR

The serum miRNA profile of PC has been well-described in several articles. Within the PC-related miRNAs, four miRNAs (miR-21, miR-17-5p, miR-155 and miR-196a) were selected as candidate miRNAs, and *RNU6B* (U6) was used as an endogenous reference. The five randomly isolated RNAs in the PC group were mixed to examine the four miRNAs and U6 by reverse transcription polymerase chain reaction (RT-PCR). Expression was considered low if the cycle threshold (CT) value was 30 or greater. Dissociation curve analysis was conducted at the end of PCR cycles to validate the specificity of the expected PCR product. After that step, the expressed miRNAs were validated in each sample by RT-PCR.

For miRNA-based RT-PCR assays, Bulge-Loop miRNA qRT-PCR Primer Sets (one RT primer and a pair of quantitative PCR primers for each set) specific for miR-21, miR-17-5p, miR-155 and miR-196a were designed by RiboBio (Guangzhou, China). Enriched small RNAs (10 μl) were reverse-transcribed using the TaqMan MicroRNA Reverse Transcription Kit (Applied Biosystems, San Diego, CA, USA) according to manufacturer’s instructions in a total reaction volume of 15 μl containing 5 μl of purified miRNAs, 1.5 μl of 10× RT buffer, 0.15 μl of 100 mM deoxyribonucleotide triphosphate, 1 μl of reverse transcriptase and 0.19 μl of RNase inhibitor (MultiScribe; Applied Biosystems), 4.16 μl of RNase-free water and 3 μl of 5× RT primer. This allowed for the creation of a miRNA cDNA library. Next, a 1:2 dilution of RT products was used as a template for real-time PCR, which was carried out on the ABI 7300 Real-Time PCR System (Applied Biosystems). The 20-μl PCR solution included 10 μl of SYBR Green Master Mix (Life Technologies, Grand Island, NY, USA), 2 μl of RT product, 2 μl of universal reverse primer, 2 μl of forward primer and 4 μl of RNase-free water. The reactions were incubated in a 96-well optical plate at 95°C for 10 min, followed by 40 cycles at 95°C for 15 s and 60°C for 1 min.

All reactions were run in triplicate. miRNA levels were quantified by measuring the value of the cycle threshold change (ΔCT) with U6 as an endogenous control (ΔCT = mean CT_miRNA_ – mean CT_U6_). Relative expression levels of the miRNAs were expressed as 2^–ΔCT^. Fold changes of miRNAs between groups were calculated by the 2^−ΔΔCT^ equation, in which ΔΔCT = ΔCT_sample_ – ΔCT_control_.

### Statistical analysis

All clinicopathologic variables and circulating miRNA expression levels were analyzed by using PASW Statistics for Windows software version 18.0 (SPSS, Chicago, IL, USA). An unpaired *t*-test was performed to compare the differences in serum miRNA levels between groups. All tests were two-sided, and *P* < 0.05 was considered statistically significant. Receiver operating characteristic (ROC) curves were established to evaluate the diagnostic value of serum miRNAs and discriminate PC patients from HPs and patients with CP or BPT.

## Results

### Circulating miRNA screening in primary adenocarcinoma patients

**Figure 1:**
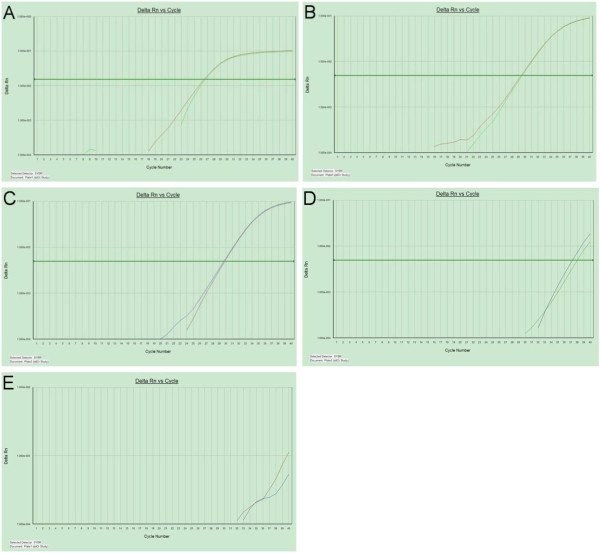
**Reverse transcription polymerase chain reaction amplification curves of microRNAs in serum of patients with pancreatic adenocarcinoma.** U6 **(A)**, miR-17-5p **(B)** and miR-21 **(C)** were stably expressed with mean cycle threshold values of 29.7, 29.4 and 26.6, respectively. miR-155 **(D)** and miR-196a **(E)** were found to have low expression levels.

**Figure 2:**
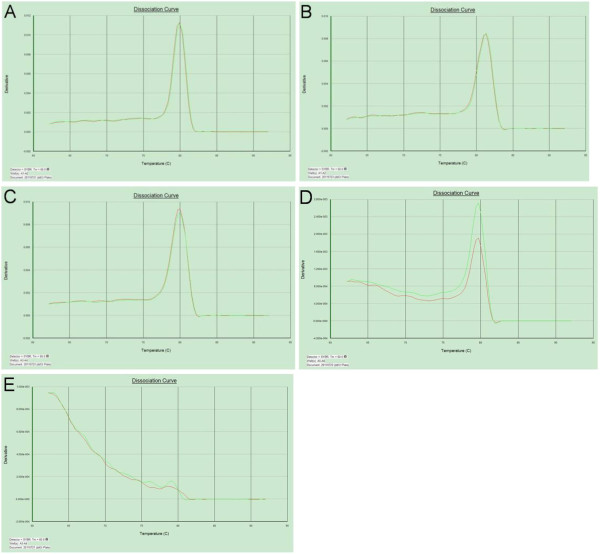
**Reverse transcription polymerase chain reaction dissociation curves of microRNAs in sera of patients with pancreatic adenocarcinoma.** Circulating U6 **(A)**, miR-17-5p **(B)** and miR-21 **(C)** miRNAs were specifically amplified with a singular peak in the dissociation curve. The variation in the dissociation curves of miR-155 **(D)** and miR-196a **(E)** indicated negative expression.

Within the PC-related miRNAs, four miRNAs (miR-21, miR-17-5p, miR-155 and miR-196a) were selected as candidate miRNAs, and RNU6B (U6) was used as an endogenous reference. Fortunately, we found that miR-21, miR-17-5P and U6 were stably expressed in the serum exosome of PC patients (CT values = 29.7, 29.4 and 26.6, respectively), whereas there was low expression of miR-155 and miR-196a (Figures 1 and 2).

### Increased expression levels of serum exosomal miR-17-5p and miR-21 in primary adenocarcinoma patients

**Figure 3:**
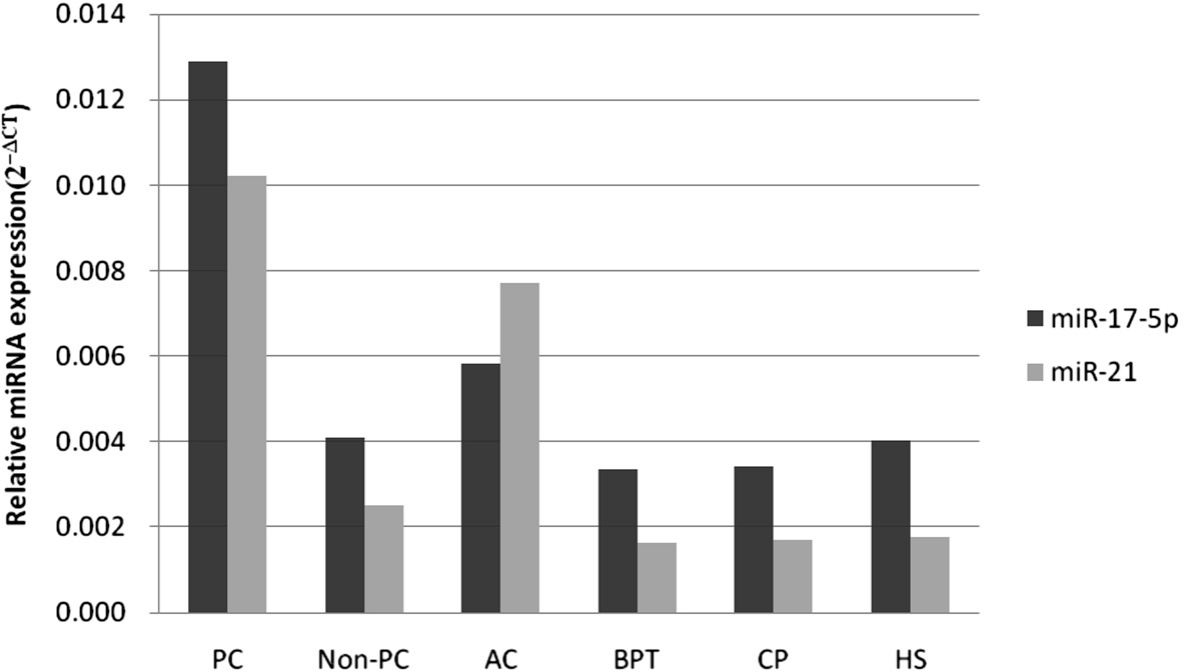
**Relative expression levels of circulating miR**-**17**-**5p and miR**-**21.** miR-17-5p and miR-21 levels were significantly higher in patients with PC than in healthy participants (HPs) and the non–primary carcinoma (non-PC) group, with 3.16- and 4.05-fold changes compared with the non-PC group and 3.2- and 5.86-fold changes compared with HPs, respectively.

The ΔCT values of miR-17-5p and miR-21 were 6.27 and 6.61 for PC patients, 7.43 and 7.02 for AC patients, 8.22 and 9.25 for BPT patients, 8.19 and 9.20 for CP patients and 7.95 and 9.16 for HPs, respectively (Table 3). The mean levels of miR-17-5p and miR-21 were significantly higher in PC patients than in HPs and the non-PC group, with 3.16- and 4.05-fold changes compared with the non-PC group and with 3.2- and 5.86-fold changes compared with HPs (Figure 3). There was no significant difference in the expression levels of miR-21 between PC patients and AC patients, although they tended to be higher in PC patients (6.61 vs. 7.02; *P* = 0.21).

**Table 3:** **Expression level of circulating miR**-**17**-**5p and miR**-**21**^**a**^

	**miR**-**17**-**5P**			**miR**-**21**		
	**ΔCT**	***t***	***P***	**ΔCT**	***t***	***P***
PC (*N* = 22)	6.27 ± 1.024			6.61 ± 0.715		
Non-PC (*N* = 27)	7.93 ± 0.784	6.386	<0.001	8.63 ± 1.239	6.781	<0.001
AC (*n* = 6)	7.43 ± 0.763	2.72	0.011	7.02 ± 0.805	1.27	0.215
BPT (*n* = 7)	8.22 ± 0.632	4.379	<0.001	9.25 ± 0.832	7.76	<0.001
CP (*n* = 6)	8.19 ± 0.932	4.119	<0.001	9.20 ± 0.784	7.698	<0.001
HP (*n* = 8)	7.95 ± 0.705	4.254	<0.001	9.16 ± 0.825	8.298	<0.001

^a^AC = ampullary carcinoma; BPT = benign pancreatic tumor; CP = chronic pancreatitis; CT = cycle threshold; HP = healthy participant; PC = primary adenocarcinoma.

### Evaluation of miR-21 and miR-17-5p as potential diagnostic markers of primary adenocarcinoma

**Figure 4:**
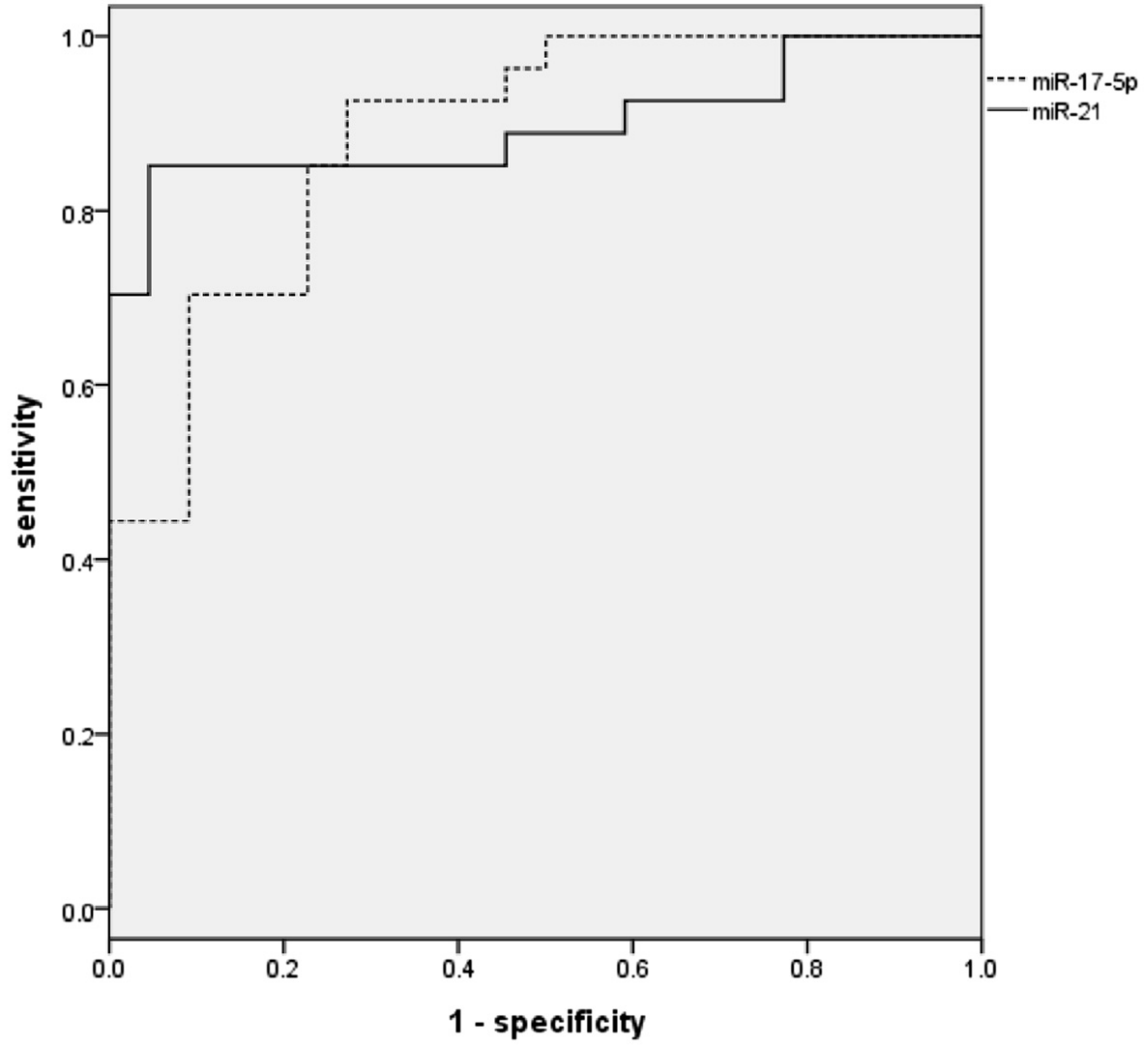
**Receiver operator characteristic curves of miR**-**17**-**5p and miR**-**21 for discriminating non**-**PC from PC.** Receiver operating characteristic areas under the curve of miR-17-5p and miR-21 were 0.887 (95% confidence interval (CI) = 0.796 to 0.978) and 0.897 (95% CI = 0.803 to 0.991), respectively. At the cutoff values of 6.826 for miR-17-5p and 7.693 for miR-21, specificities and sensitivities were 72.7% and 92.6% for miR-17-5p and 95.5% and 81.5% for miR-21, respectively.

ROC curve analyses were performed to evaluate the diagnostic value of miR-21 and miR-17-5p for PC. These analyses revealed that the levels of serum miR-17-5p and miR-21 were potential markers for discriminating non-PC from PC, with ROC areas under the curve of 0.887 (95% confidence interval (CI) = 0.796 to 0.978) and 0.897 (95% CI = 0.803 to 0.991), respectively (Figure 4). At the cutoff values of 6.826 for miR-17-5p and 7.693 for miR-21, PC diagnostic sensitivities and specificities were 72.7% and 92.6% for miR-17-5p and 95.5% and 81.5% for miR-21, respectively (Table 4).

**Table 4:** **Cycle threshold change values of circulating miR**-**17**-**5p and miR**-**21 predicting primary adenocarcinoma**^**a**^

	**miR-17-5P**		**miR-21**	
	**≤6.826**	**>6.826**	**≤7.693**	**>7.693**
PC (*N* = 22)	16	6	21	1
Non-PC (*N* = 27)	2	25	5	22
χ^2^	22.3		28.8	
OR (95% CI)^a^	4.6 (2.2 to about 9.6)		18.6 (2.7 to about 127.5)	
*P* value	<0.001		<0.001	

^a^CI = confidence limits; OR = odds ratio; PC, primary adenocarcinoma.

### Relationship between miR-17-5p, miR-21 and clinicopathologic features of primary adenocarcinoma

**Figure 5:**
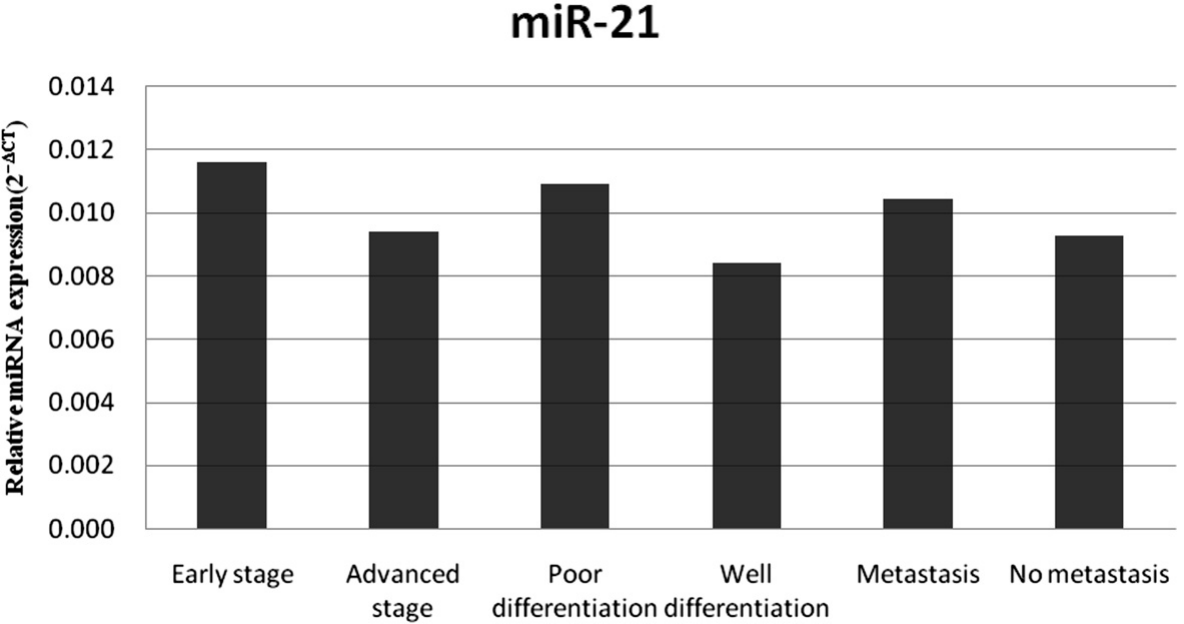
**Relative expression level of circulating miR**-**21 in each group.** On the basis of RT-PCR assay of miR-21, there were 1.23-, 1.28- and 1.13-fold changes between the different stage, differentiation and metastasis groups of PC patients (*P* = 0.339, 0.385 and 0.668, respectively), indicating no significant correlations with stage, differentiation or metastasis.

**Figure 6:**
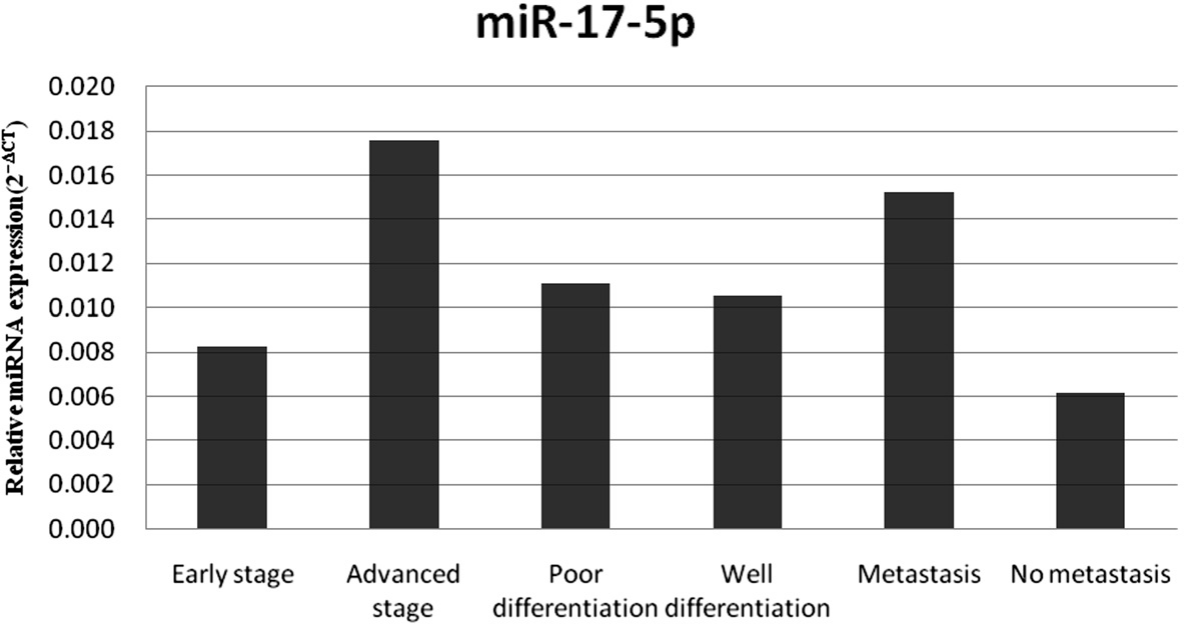
**Relative expression level of circulating miR**-**17**-**5p in each group.** Circulating miR-17-5p was significantly higher in the advanced stage and metastasis groups, with 2.13-fold (*P* = 0.01) and 2.46-fold (*P* = 0.017) changes, respectively. No significant difference was found between the poorly differentiated and well-differentiated groups.

On the basis of RT-PCR assays of miR-21, we found that there were 1.23-, 1.28- and 1.13-fold changes among the different stage, differentiation and metastasis groups of PC patients (*P* = 0.339, 0.385 and 0.668, respectively), indicating no significant correlations with stage, differentiation or metastasis (Figure 5). Circulating miR-17-5p was significantly elevated in the group of advanced stage and metastatic PC patients, with 2.13-fold (*P* = 0.01) and 2.46-fold (*P* = 0.017) changes, respectively, but it seems that it did not correlate with tumor differentiation in patients with PDAC (Figure 6). There were eight (88.9%) of nine and two (15.4%) of thirteen resectable PC cases in the early stage and advanced stage groups, respectively (Table 5), indicating that elevated serum exosomal miR-17-5p was a potential biomarker for unresectable PC.

**Table 5:** **Cross-table of primary adenocarcinoma stage with resectability**^**a**^

**Stage**	**Resectable**	**Unresectable**	**Total**
Early stage	8	1	9
Advanced stage	2	11	13
Total	10	12	22

^a^χ^2^ = 11.589, *P* = 0.002.

## Discussion

Early detection of PC remains a challenge for clinical scientists. More than 80% of patients with PC who were diagnosed with advanced stage disease could not be cured by surgical resection in a previous study [10]. In addition, CP and other BPT (for example, islet cell tumor, solid pseudopapillary tumor), which present with clinical and radiologic characteristics similar to those of PC, such as jaundice, weight loss, elevated carbohydrate antigen 19-9 (CA 19-9) level and the presence of diffuse pancreatic enlargement or a focal pancreatic mass must be distinguished from PC to avoid unnecessary surgery. Currently available tumor markers of PC, carcinoembryonic antigen (CEA) and CA 19-9, exhibit low sensitivity and specificity for PC diagnosis, and they are particularly unsatisfactory for early detection of this disease. Although novel blood-based biomarker discoveries (for example, using proteomic technologies) have shown promise for cancer detection [11], new approaches that can complement and improve upon current strategies are still urgently needed. There are increasing reports of circulating miRNAs as potential biomarkers for PC detection [12]. Compared with other large molecules such as mRNA, miRNAs have been found to be stably expressed in human peripheral blood and resistant to DNase or RNase activity [13], whereas mRNA has been found to be rapidly degraded by RNase A.

Exosomes, which can be released by many different cells to the extracellular microenvironment, are small membrane vesicles of about 60 to 100 nm containing protein, mRNA and miRNA. Exosomes have been shown to participate in the immune system by mediating antigen presentation and transporting miRNA. Previous miRNA profile studies have suggested that circulating miRNAs are preferable for cancer detection, including PC [14,15]. However, there is still a lack of serum exosomal miRNA labeling of PC patients. In this study, we examined four candidate miRNAs in the serum exosome of PC and non-PC patients and analyzed the relationships between the expression levels and clinical pathological features of disease to assess their clinical application for diagnosing and monitoring PC. We found that miR-17-5p and miR-21 were significantly elevated in the serum exosome of PC patients. ROC curve analysis revealed that serum exosomal miR-21 and miR-17-5p were sensitive biomarkers for differentiating PC from CP and BPT with high sensitivity and specificity. Importantly, our data also demonstrate that higher serum exosomal miR-17-5p was associated with advanced stage and metastasis of PC, which closely correlated with the resectability of PC tumors. We believe that the identification of miR-17-5p may represent a key advancement in research on valuable serum biomarkers that have the potential to indicate unresectable PC. Because serum miR-21 has been reported to be significantly overexpressed in patients with other types of malignant tumors, including gastric cancer [15], breast cancer [16,17], ovarian cancer [18], colon cancer [19] and hepatic cancer [20], it may not be a specific biomarker for PC patients.

Apart from the potential of serum exosomal miRNA levels to aid in clinical diagnosis and prognosis prediction, this study also revealed some intriguing aspects regarding their potential function in PC. miR-21 is overexpressed in various human tissues, including PC [21] and other types of cancer [22]. In the present study, we also found serum miR-21 was increased by 5.86-fold in patients with PC compared with HPs, suggesting that miR-21 is a universal serum biomarker for several cancers and indicating that miR-21 may be an important regulatory molecule in carcinogenesis. Furthermore, the serum exosomal miR-17-5p level increased with PC progression, which is consistent with a previous study of PC tissue [23]. It is questionable whether serum exosomal miR-17-5p serves as an important intercellular messenger mediating PC invasion and metastasis. As the functional roles of miR-17-5p in tumor biology are unrevealed, we postulate that exosomal miR-17-5p that predicts a poor prognosis for PC patients may be involved in the inflammatory response or tumor immunologic escape via the exosome pathway [24]. Further studies including a serum exosomal miRNA profile of PC and function analysis will be performed to explore these exciting hypotheses.

miRNA assay has a few advantages over conventional blood-based biomarker examinations such as proteomic technologies and mRNA assays. (1) Circulating miRNA expression profiles vary from those in blood cells, some of which may represent special tissue or cancer origin [25]. (2) miRNAs were expressed with higher stability in blood compared with mRNA and protein. Harsh environments, such as boiling, low or high pH, extended storage and freeze–thaw cycles, had minimal effect on miRNA expression levels as measured by RT-PCR [23,26]. (3) Exosomal miRNAs are abnormally secreted by cancer cells, and those secreted miRNAs may represent a class of signaling molecules in mediating intercellular communication [27]. These advantages show the potential value of using serum exosomal miRNAs as biomarkers for cancer detection.

## Conclusions

Our study demonstrates that serum exosomal miR-17-5p and miR-21 levels were significantly elevated in PC patients compared with HPs and non-PC patients, suggesting that these serum exosomal miRNAs may serve as potential biomarkers of PC, especially serum miR-17-5p, which has seldom been reported in other types of cancer, except for one report in gastric cancer [12]. The relationship between high levels of miR-17-5p with PC metastasis and advanced stage encourage us to perform further research to identify the biological function of exosomal miR-17-5p as an intercellular messenger mediating PC invasion and metastasis.

## Competing interests

The authors have no financial interest with regard to any product or concept discussed in this article. The authors declare that they have no competing interests.

## Authors’ contributions

RQ: was the main executant of the study. He collected the clinical data of all patients and healthy participants, provided serum samples for the study and participated in the statistical analysis. GD: was a laboratory technician. He isolated exosomal RNA from serum, carried out the examination of RT-PCR, made the statistic analysis and drafted the manuscript. JC: is a graduate student. He helped Dr. Ding to perform the experimental examinations. LC: is the leader of the research team. He designed the study, checked all data and provided technological supports for the study. All authors read and approved the final manuscript. The first two authors had equal contributions to the study.
